# Maladie de Takayasu et polyarthrite rhumatoïde: une association rare - à propos d'une observation

**Published:** 2012-07-03

**Authors:** Faten Frikha, Fatma Maazoun, Mouna Snoussi, Leila Abid, Hanen Abid, Walid Bouassida, Neila Kaddour, Zouhir Bahloul

**Affiliations:** 1 Service de Médecine interne, CHU Hédi Chaker, 3029 Sfax, Tunisia; 2 Service de Cardiologie, CHU Hédi Chaker 3029 Sfax, Tunisia; 3 Service de Radiologie, CHU Habib Bourguiba 3029 Sfax, Tunisia; 4 Service d'ophtalmologie, CHU Habib Bourguiba, 3029 Sfax, Tunisia

**Keywords:** Maladie de Takayasu, polyarthrite rhumatoïde, aortite, risque cardiovasculaire

## Abstract

L'artérite de Takayasu ou maladie de Takayasu (MT) et la polyarthrite rhumatoïde (PR) et sont deux maladies inflammatoires chroniques et leur association a été rapportée dans la littérature à travers quelques observations de cas sporadiques. Nous rapportons une nouvelle observation d'une telle association. Une patiente âgée de 44 ans, diagnostiquée avec une polyarthrite rhumatoïde à facteur rhumatoïde positif, qui a développé des céphalées avec des vertiges de caractère permanent. L'examen révélait un pouls radial et huméral abolis à droite, un souffle carotidien bilatéral et une tension artérielle imprenable à droite. L'artériographie a confirmé la présence d'une atteinte de l'arc aortique type MT. Le diagnostic d'une maladie de Takayasu associée à une polyarthrite rhumatoïde était retenu. La patiente était traitée par une corticothérapie (prednisone à la dose de 0,5 mg/kg par jour) et un traitement de fond par Méthotrexate avec une bonne réponse initiale. A travers notre observation et une revue de la littérature, les caractéristiques épidémiologiques, étiopathogéniques, cliniques, thérapeutiques et évolutives de cette association seront discutées.

## Introduction

La maladie de Takayasu est une artérite inflammatoire rare, plus fréquente chez la femme, qui affecte les vaisseaux de gros calibres (l'aorte thoracique et ses branches, le tronc artériel brachiocéphalique et les artères sous-clavières, ainsi que les artères pulmonaires et coronaires) et dont l’étiologie reste inconnue. Ses mécanismes physiopathologiques sont probablement plurifactoriels, associant des facteurs génétiques et immunologiques. Elle peut être associée à plusieurs pathologies auto-immunes (entérocolopathies principalement la maladie de Crohn, spondylarthrite ankylosante, Lupus érythémateux systémique, sarcoïdose…) [1, 2] L'association de la maladie de Takayasu à une polyarthrite rhumatoïde est rare [3]. Nous en rapportons une nouvelle observation et discuterons les particularités de cette association à travers une revue de la littérature.

## Patient et observation

Une femme âgée de 44 ans, d'origine tunisienne, sans antécédents pathologiques, était hospitalisée dans le service en 1997 pour une polyarthrite chronique associée à des vertiges. A l'admission, l'interrogatoire révélait la notion de polyarthralgies inflammatoires évoluant depuis trois ans avec des épisodes d'arthrites touchant de façon bilatérale et symétrique les poignets, les articulations métacarpo-phalangiennes (MCP) et interphalangiennes (IPP) proximales des mains, les chevilles et les articulations métatarso-phalangiennes, avec réveils nocturnes multiples et dérouillage matinal de 2 heures. La patiente signalait aussi la notion de céphalée et de vertige avec éclipse visuelle évoluant de façon paroxystique depuis quelques mois. A l'examen, il y'avait une synovite des MCP des deux mains, une ankylose du poignet droit et une limitation de la mobilité des deux chevilles. L'examen cardio-vasculaire révélait un pouls radial et huméral absents à droite et une tension artérielle imprenable à droite. L'auscultation cardiaque et des trajets vasculaires trouvait un souffle systolique au foyer aortique et un souffle au niveau des vaisseaux du cou. L'examen neurologique et l'examen ophtalmologique étaient normaux. Le reste de l'examen était sans anomalies.

A la biologie, la vitesse de sédimentation était à 55 mm à la 1ère heure, la protéine C réactive était à 6 mg/l. La numération formule sanguine révélait une anémie à 9,9 g/dl d'hémoglobine microcytaire avec ferritinémie normale. Les bilans hépatique et rénal étaient normaux. La sérologie rhumatoïde était fortement positive en Latex et Waaler Rose. Les AAN étaient positifs à 1/320. Les anticorps antiphospholipides ainsi que la cryoglobulinémie étaient négatifs. Les anti cytoplasme des polynucléaires neutrophiles et les marqueurs des hépatites virales étaient négatifs. L'enquête tuberculeuse était négative.

**Figure 1:**
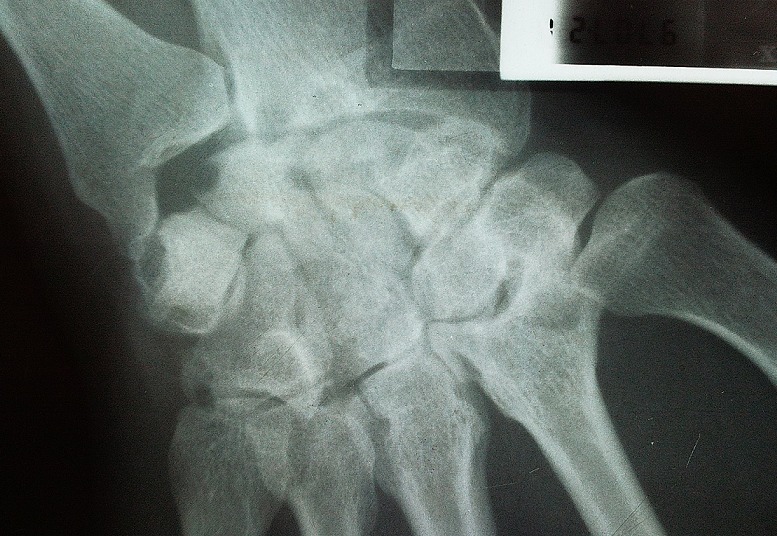
Radiographie de la main droite: carpite droite stade 3 (érosions avec pincement des os du carpe)

**Figure 2:**
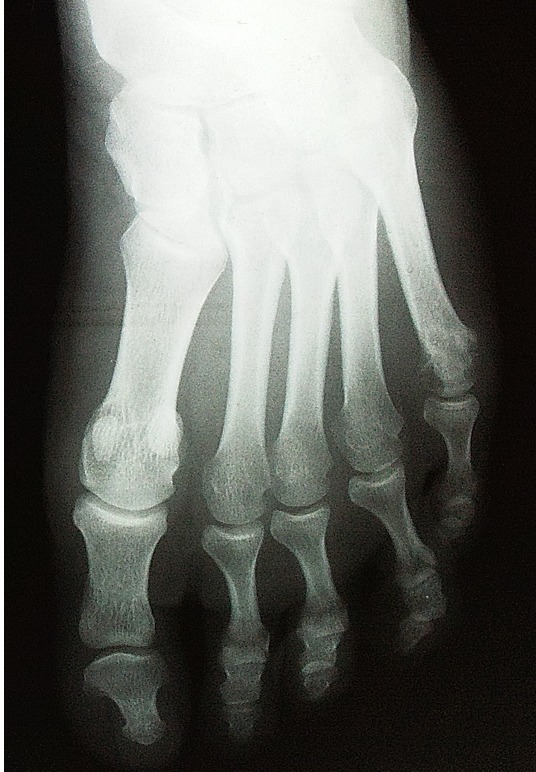
Radiographie du pied gauche face: géodes de la tête du 5ème métatarsien gauche

La radiographie des mains révélait une carpite radiologique stade 3 de Steinbrocker à droite et des images géodiques au niveau du 2ème métacarpien et du cubitus gauche. La radiographie des pieds montraient de multiples géodes au niveau de la tête du 5ème métatarsien gauche (Figure 1, Figure 2). L’échographie doppler des Troncs supra-aortiques (TSA) trouvait un épaississement pariétal diffus des axes carotidiens et de leurs bifurcations, une asymétrie du flux vertébral et sous clavier plus bas à droite.

**Figure 3:**
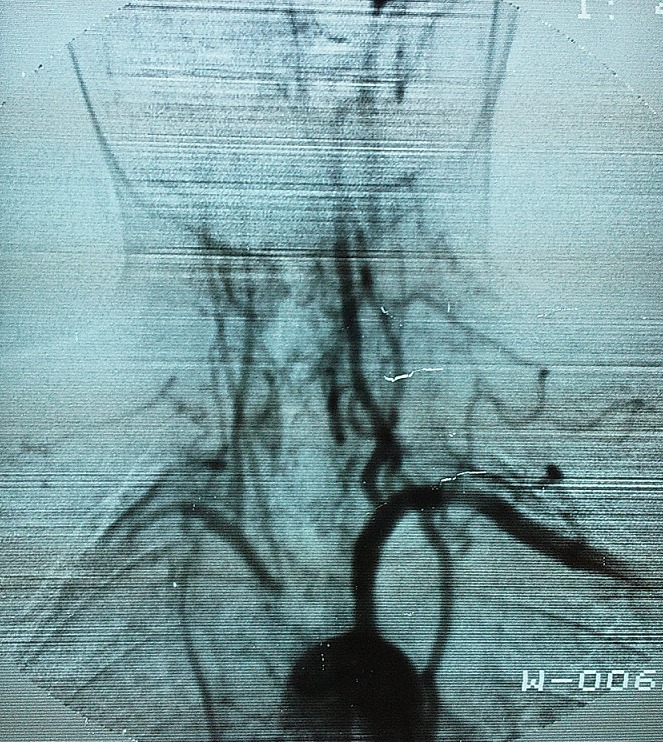
Artériographie: sténose proximale du tronc brachio-céphalique et de la sous-clavière droite

**Figure 4:**
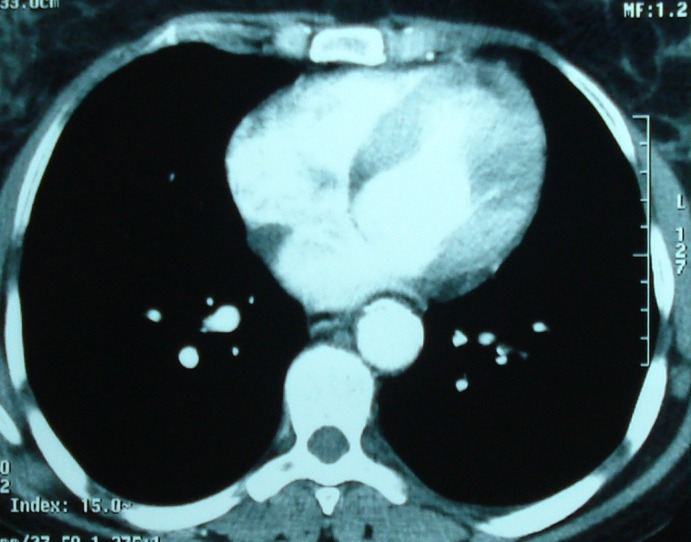
Angio-scanner de l'aorte: épaississement pariétal et circonférentiel calcifié de l'aorte thoracique descendante

L’écho-Doppler des membres supérieurs révélait une asymétrie franche du débit artériel des membres supérieurs avec un tracé plus amorti des résistances périphériques diminuées de façon diffuse à droite. L'artériographie de l'aorte thoracique et abdominale révélait un aspect filiforme de la lumière des artères carotides primitives droite et gauche ainsi que des vertébrales et des segments distaux de la sous Clavière droite en rapport avec un épaississement en manchon caractéristique de la maladie de Takayasu et une sténose proximale du tronc artériel brachio-céphalique coté à 50% à 1,5 cm de son origine (Figure 3). L'aspect de l'aorte thoracique, de l'aorte abdominale, des artères rénales et iliaques était normal. La radiographie thoracique et l’échographie cardiaque étaient sans anomalies. Le diagnostic d'une polyarthrite rhumatoïde était retenu selon les critères de l'ACR [4]. Cette PR était associée à une maladie de Takayasu retenue selon les critères clinico-radiologiques de l'American College of Rheumatology [5] classée stade I d'Ishikawa. La patiente était traitée pour sa PR par des AINS et un traitement de fond à base de Méthotrexate à la dose de 10 mg/semaine. Concernant la maladie de Takayasu, il s'agissait d'une maladie active avec syndrome inflammatoire biologique et des lésions d’épaississement vasculaire à l'imagerie. La patiente était mise sous corticothérapie à la dose de 0.5 mg/Kg/jour pendant 6 semaines puis dégressive jusqu’à un palier de 10 mg par jour, associée à une anti agrégation plaquettaire. L’évolution était initialement favorable sur le plan articulaire et vasculaire. En 2001, la patiente présentait une poussée de sa maladie de Takayasu avec atteinte aortique à type d'une insuffisance aortique modérée sans aucun retentissement et une occlusion de l'artère carotide primitive gauche nécessitant sa mise sous corticothérapie à forte dose (prednisone 1 mg/Kg/jour) pendant une durée de 5 semaines. En 2003, la patiente développait une hypertension artérielle (HTA) grade 1 sans retentissement viscéral. L’écho-doppler des artères rénales était normale. Cette HTA était rattachée à la sténose serrée des Troncs supra-aortiques. Elle était jugée bénéfique pour la perfusion cérébrale et donc n'ayant pas nécessité le recours à un traitement antihypertenseur. En 2011, la patiente se présentait avec une épisclérite récidivante de l’œil droit non améliorée par le traitement local et les anti-inflammatoires non stéroidiens. Cette épisclérite avait justifié sa mise sous corticothérapie à dose moyenne (prednisone 0,5 mg/Kg/jour) pendant une durée de 3 semaines avec une dégression progressive. L’évolution était favorable. Après un recul évolutif de 13 ans et 8 mois, la polyarthrite rhumatoïde parait stable et modérément évolutive avec un score d'activité DAS28 à 3,56. Le facteur rhumatoïde était positif à 531 UI/ml. Les anticorps anti CCP étaient positifs aussi. Afin d’évaluer l'aortite, un angio-scanner thoracique et abdominal était réalisé. Il révélait un épaississement pariétal circonférentiel calcifié régulier de la paroi de l'aorte thoracique dans ses segments I et II au niveau de l’émergence des TSA, avec un épaississement pariétal et circonférentiel calcifié par endroit de l'aorte thoracique descendante dans ces différents segments (Figure 4).

## Discussion

La maladie de Takayasu (MT) est une aorto-artérite inflammatoire granulomateuse non nécrosante atteignant les vaisseaux de gros et de moyens calibres (l'aorte et ses branches principales ainsi que les artères pulmonaires). Contrairement à la polyarthrite rhumatoïde, qui est un rhumatisme inflammatoire fréquent dans le monde et dans notre pays (prévalence estimée entre 0,3 et 0,8% de la population adulte), la maladie de Takayasu constitue une pathologie rare, avec une fréquence importante en Asie du Sud-est, en Inde et en Amérique du Sud. Elle touche essentiellement l'adulte jeune, avec une incidence maximale au cours de la deuxième ou troisième décennie [6, 7]. Cependant, et comme pour la polyarthrite rhumatoïde (4 fois plus fréquente chez la femme), il existe une prédominance féminine marquée de la MT, avec un sex-ratio femme/homme compris entre 2 et 15 selon les séries [6, 7].

Plusieurs études ont démontré que la MT peut être associée à des troubles immunitaires ou des maladies systémiques ou granulomateuses, telles que: les entérocolopathies inflammatoires (maladie de Crohn principalement, rectocolite hémorragique) la spondylarthrite ankylosante, et plus rarement le lupus érythémateux disséminé, la sarcoïdose [1–3]. Ces associations sont exceptionnelles mais semblent être non fortuites. Le développement simultané d'une PR et d'une MT est aussi rare, comme dans notre observation (un seul cas parmi une cohorte de 37 cas de maladie de Takayasu colligés entre 1996 et 2010). La revue de la littérature (1961–2007) rapporte une vingtaine d'observations de cette association [3, 8–17]. Dans la revue faite par Korkmaz et al [3], l'auteur rapportait 20 cas. Il s'agissait de 14 femmes et 6 hommes d’âge moyen égal à 55,5 ans (extrêmes 20 et 82 ans). L’âge de survenue de cette association est plus élevé que celui de la MT isolée [18]. Le délai moyen entre le développement de la PR et la survenue de la MT était de 7,5 ans. La survenue de la PR précède celle de la MT dans la majorité des cas. Ceci serait expliqué par le fait que les symptômes initiaux de la MT, et entre autre les arthralgies, soient pris sur le compte de manifestations liées à la PR [17]. Sur les 20 cas étudiés, la maladie de takayasu a précèdé la survenue de la PR dans un seul cas. Il s'agit d'une femme ayant développé une MT et une PR à 26 et 37 ans respectivement [11].

Les données étudiant les bases étiopathogéniques de cette association sont rares. Le caractère fortuit ou non de l'association de ces 2 pathologies ne peut pas être alors confirmé, surtout que certaines analogies pathogéniques rapprochent ces deux affections. L’étiologie de la MT reste encore inconnue mais l'auto-immunité semble jouer un rôle dans sa physiopathologie [3, 17] avec intervention de l'immunité cellulaire dans la constitution des lésions artérielles [6]. La coexistence simultanée de ces maladies semble indiquer que l′apparition de la MT peut être liée à une dysrégulation du système immunitaire. En fait, dans les 2 maladies, l'activation des Lymphocytes T et des macrophages par des stimuli endogènes ou exogènes, aboutit à la production des cytokines pro-inflammatoires (IL-1, IL-6, TNF), responsables de l'altération de l’état général et de l’élévation des protéines de l'inflammation. Certains auteurs rapportent des facteurs génétiques dont le rôle pathogénique reste prépondérant comme le système human leucocyte antigen (HLA). Certains haplotypes du système HLA sont surreprésentés chez les patients atteints de PR (HLA Classe II: DRB10401, DRB1*0404, DRB1*0101) et chez les patients atteints de MT (HLA de classe II (HLA-DRB1*1501, DRB5*0102, DQA1*0103, DQB1*0601, DPA1*02, DPB1*0401), ainsi que de certains allèles HLA de classe I (HLA-B5201, HLA-B39) [19]. Chez notre patiente, la recherche d'haplotypes HLA n'a pas été réalisée.

Selon certains auteurs, l'association PR-MT reste à discuter puisque l′aortite et des troubles de la valve aortique sont parfois observés au cours de la PR [15, 20]. En effet, Selon Gravallese et al [14], l′autopsie a révélé une aortite ou une inflammation valvulaire dans 15% des patients atteints de PR sans signes d′insuffisance aortique. D'autre part, la fréquence des arthralgies est élevée au cours de la MT. Sato et al [21] ont rapporté que des arthralgies ou des arthrites ont été observées chez 26% de leurs patients ayant une Maladie de Takayasu. Hall et al ont indiqué que des arthralgies ou des synovites ont été notées dans 56% et 22% des 32 patients avec une MT, respectivement [22].

Sur le plan clinique, on distingue au cours de la MT deux phases évolutives: une première phase dite systémique préocclusive caractérisée par des signes généraux peu spécifiques (fièvre, amaigrissement, arthromyalgies, signes ophtalmologiques à type d’épisclérite ou d'uvéite, ou signes cutanés à type d’érythème noueux ou hypodermite nodulaire) et une deuxième phase occlusive o[ugrave] s'installent les conséquences ischémiques de l'atteinte artérielle (claudication intermittente, souffle vasculaire, asymétrie tensionnelle ou de modification de pouls). La maladie de Takayasu dans sa phase occlusive est certaine chez notre patiente avec des lésions artérielles typiques objectivées par l'artériographie, d'autant plus que l'atteinte des artères vertébrales et sousclavières, retrouvée dans notre observation, est extrêmement fréquente au cours de la MT [7]. D'autres diagnostics différentiels d'aortites telles que les aortites infectieuses (syphilis et tuberculose), les aortites inflammatoires (lupus, polyarthrite rhumatoïde, spondylarthropathies, maladie de Behçet, maladie de Kawasaki et artérite à cellules géantes) et l'athérosclérose ont été aussi éliminées chez notre patiente [20, 23]. En fait, la mise en évidence en écho-Doppler d'un épaississement pariétal au niveau carotidien et sous-clavier, dont le caractère est homogène, circonférentiel s'oppose aux lésions d'athérosclérose, est en faveur du diagnostic de maladie de Takayasu [20].

Le diagnostic de la polyarthrite rhumatoïde est aussi certain chez notre patiente, puisque une polyarthrite érosive avec un facteur rhumatoïde fortement positif est exceptionnelle au cours de l'artérite de Takayasu. L’éventualité que l'atteinte aortique soit rattachée à la PR est aussi peu probable. La polyarthrite rhumatoïde se complique rarement d'aortite, et cette complication est généralement l'apanage des polyarthrites sévères chroniques avec vascularite, ce qui n'est pas le cas pour notre patiente [20]. Gravallese et al. ont observé 10 aortites sur 188 autopsies consécutives de patients ayant une polyarthrite rhumatoïde [14]. La polyarthrite rhumatoïde évoluait depuis environ 9 ans. L'aorte thoracique était la plus fréquemment atteinte. Deux patients avaient un anévrisme de l'aorte thoracique et un de l'aorte abdominale. Histologiquement, il y avait une nécrose des cellules musculaires lisses de la média et un infiltrat inflammatoire principalement lymphoplamocytaire. Des granulomes rhumatoïdes étaient observés dans la paroi aortique chez 5 patients. Sept patients sur 10 avaient une vascularite rhumatoïde atteignant en moyenne 10 organes, responsable du décès chez 6 patients.

Sur le plan biologique, les deux maladies se caractérisent par un syndrome inflammatoire non spécifique avec accélération de la VS et élévation de la C-réactive protéine, du fibrinogène. La vitesse de sédimentation est souvent utilisée comme marqueur de suivi de l'activité de la maladie de Takayasu. Cependant, le meilleur marqueur de l'activité de cette vascularite semble être l'apparition de nouvelles lésion ce qui donne toute l'importance à la répétition des examens d'imagerie vasculaire. L’évaluation de l'activité de la MT chez notre patiente s'est basée essentiellement sur la réalisation d'un angio-scanner ayant mis en évidence un aspect d’épaississement circonférentiel régulier calcifié de l'ensemble de l'aorte, témoignant de lésions anciennes et d'un stade chronique tardif inactif de la maladie, plutôt que de lésions d'athérosclérose. Cette technique a montré son intérêt dans le suivi thérapeutique de ces patients [24].

En l'absence de marqueurs spécifiques, le diagnostic de maladie de Takayasu est basé sur un faisceau d'arguments essentiellement cliniques et radiologiques mettant en évidence une atteinte non athéromateuse de l'aorte et de ses branches proximales. Plusieurs types de critères diagnostiques se sont développés mais ne sont pas toujours d'application facile (Fiessinger 1982, Ishikawa 1988, ACR, Blétry, Sharma).Pour notre malade, nous nous sommes basées sur les critères de l'ACR utilisant l'artériographie comme moyen d'imagerie de diagnostic de référence. Nous avons eu recours à l'angio-scanner pour le suivi thérapeutique.

Le pronostic de l'artérite de Takaysu dépend essentiellement de l'agressivité initiale de la maladie et de ses complications (hypertension, anévrismes et insuffisance aortique). La mortalité est d'environ 10% à dix ans et est corrélée au nombre d'atteintes viscérales graves Le risque de rechute est important, touchant près de 20%des patients [25]. L'hypertension artérielle (HTA) est fréquente au cours de la MT, retrouvée dans 33 à 72% des cas dans la littérature. Les mécanismes incriminés dans la genèse de cette HTA sont multiples. Il peut s'agir d'une sténose de l'aorte sus-rénale, ou d'une HTA rénovasculaire par sténose des artères rénales. Les anomalies des barorécepteurs, la baisse de la compliance aortique, l'augmentation des résistances périphériques, l'insuffisance aortique, la baisse du flux sanguin cérébral et la corticothérapie utilisée au cours de cette vascularite systémique, peuvent expliquer certaines hypertensions artérielles sans lésions vasculaires sous-jacentes responsables, dont la prévalence reste élevée par rapport à une population générale dans la même tranche d’âge [26].

La mise en évidence d'une HTA au cours de la MT doit systématiquement conduire à la recherche d'une sténose des artères rénales, retrouvée dans près de 50 à 70% des cas. Cette recherche était normale chez notre patiente et l'HTA était rattachée plutôt à la sténose serrée des Troncs supra-aortiques. Le risque cardiovasculaire est aussi augmenté au cours de la PR qui est considéré par certains auteurs comme un facteur de risque cardiovasculaire aussi important que le diabète. La pathologie cardiovasculaire semble être la cause principale de l'augmentation de la mortalité au cours de ce rhumatisme inflammatoire chronique [27]. Le pronostic chez notre malade semble être lié aux conséquences vasculaires de ces 2 maladies. En effet, le risque cardiovasculaire est majoré chez notre patiente par l'association de ces deux pathologies avec une augmentation du risque important pour les cardiopathies ischémiques et pour les accidents ischémiques cérébraux.

La maladie de Takayasu est une maladie qui nécessite souvent des traitements médicaux et chirurgicaux lourds. Ces dix dernières années, certains immunosuppresseurs ont fait la preuve de leur efficacité chez des malades présentant une corticodépendance ou une corticorésistance. L'utilisation de méthotrexate permet d'obtenir près de 80% de rémission avec une bonne tolérance, mais avec un fort taux de rechutes à l'arrêt du traitement. L'utilisation du méthotrexate était indiqué d'emblée pour la polyarthrite rhumatoïde avec un effet certainement additif pour la MT.

## Conclusion

L'association d'une maladie de Takayasu et d'une polyarthrite est rare. Certaines analogies pathogéniques peuvent rapprocher ces deux affections. Cette association ne semble pas modifier l’évolution naturelle des deux pathologies mais le risque cardio-vasculaire est sans doute majoré. Enfin, notre observation souligne l'importance de réaliser un examen clinique vasculaire complet chez les patients porteurs d'une spolyarthrite rhumatoïde, afin de rechercher une artérite inflammatoire associée sous-jacente, jusque là asymptomatique.
